# Thermographic skin temperature measurement compared with cold sensation in predicting the efficacy and distribution of epidural anesthesia

**DOI:** 10.1007/s10877-017-0026-y

**Published:** 2017-05-15

**Authors:** Arnoud A. Bruins, Kay R. J. Kistemaker, Annemieke Boom, John H. G. M. Klaessens, Rudolf M. Verdaasdonk, Christa Boer

**Affiliations:** 1 Department of Anesthesiology, Institute for Cardiovascular Research, VU University Medical Center, De Boelelaan 1117, 1081 HV Amsterdam, The Netherlands; 2 Spaarne Gasthuis, Spaarnepoort 1, 2134 TM Hoofddorp, The Netherlands; 3 Department of Physics and Medical Technology, Institute for Cardiovascular Research, VU University Medical Center, De Boelelaan 1117, 1081 HV Amsterdam, The Netherlands

**Keywords:** Thermography, Epidural anesthesia, Postoperative pain, Cold sensation test

## Abstract

Due to the high rates of epidural failure (3–32%), novel techniques are required to objectively assess the successfulness of an epidural block. In this study we therefore investigated whether thermographic temperature measurements have a higher predictive value for a successful epidural block when compared to the cold sensation test as gold standard. Epidural anesthesia was induced in 61 patients undergoing elective abdominal, thoracic or orthopedic surgery. A thermographic picture was recorded at 5, 10 and 15 min following epidural anesthesia induction. After 15 min a cold sensation test was performed. Epidural anesthesia is associated with a decrease in skin temperature. Thermography predicts a successful epidural block with a sensitivity of 54% and a PPV of 92% and a specificity of 67% and a NPV of 17%. The cold sensation test shows a higher sensitivity and PPV than thermography (97 and 93%), but a lower specificity and NPV than thermography (25 and 50%). Thermographic temperature measurements can be used as an additional and objective method for the assessment of the effectiveness of an epidural block next to the cold sensation test, but have a low sensitivity and negative predictive value. The local decrease in temperature as observed in our study during epidural anesthesia is mainly attributed to a core-to-peripheral redistribution of body heat and vasodilation.

## Introduction

Epidural anesthesia is widely used for perioperative pain management in major abdominal, thoracic and orthopedic surgery. Epidural anesthesia attenuates the surgical stress response and provides superior analgesia in the early postoperative period. However, due to inaccurate placement, epidural anesthesia is ineffective in 3–32% of the cases [1–6]. A crucial step in improving the success rate of epidural anesthesia is preoperative confirmation of the correct placement of the epidural block with an objective, fast and real-time method.

The most widely used method for the assessment of the efficacy of epidural anesthesia is the cold sensation test [6]. During this test, patients are assessed for cold sensation in the thoracic to sacral dermatomes bilaterally to determine which area of the body is anesthetized [6]. However, this type of sensory block testing is subjective, time consuming, depending on patient cooperation and may be associated with false positive and false negative results [6, 7].

A thermographic image is a matrix of temperatures with a standard color code (e.g. iron or rainbow), which represent the surface temperature of the imaged skin or tissue [8–14].

Local anesthetics used for epidural anesthesia block the local sympathetic nerves, which subsequently cause vasodilatation and the blood flow will increase. Resulting in a local temperature change of the skin [15]. These temperature changes can be assessed by thermography, as was previously shown in patients undergoing an axillary regional blockade for hand or forearm surgery [16]. They found that the thermographic camera showed higher combined values for sensitivity, specificity and positive and negative predictive values compared to cold sensation testing [16]. Moreover, Klaessens et al. showed that thermography revealed distinct skin temperature differences that were well correlated with the areas of the peripheral nerve block [17].

The absence of a painful stimulus in the preoperative setting creates a diagnostic dilemma in the detection of epidural failure. In this study we aimed to investigate whether thermographic temperature measurement can be used as a method of assessing the efficacy and distribution of epidural anesthesia, and whether this type of measurement has a higher predictive value for a successful epidural block when compared to the cold sensation test as gold standard.

## Methods

### Subjects

This observational study was approved by the Human Subjects Committees of the VU Medical Center and Spaarne Gasthuis (METc 15-332). Written informed consent was obtained from all participants. Patients were included in case of an American Society of Anesthesiology physical status I–III in the age range of 46 to 91 years old, and scheduled for elective abdominal, thoracic or orthopedic surgery with an indication for epidural anesthesia. The epidural catheter was placed by the anesthesiologist in the preoperative care unit and general anesthesia was provided during surgery.

Exclusion criteria comprised withdrawal of consent, contra-indications for epidural anesthesia, psychiatric disorders, or drug abuse. Patients with an infectious disease, inflammation, eczema or with burns of the skin in the region of the insertion of the epidural catheter were also not included.

The surrounding temperature in the anesthesia department was kept at a constant level at 21 °C with a constant relative humidity of the surrounding air. Prior to examination, patients were undressed for at least 30 s to adjust to the surrounding temperature. After the thermographic imaging was performed, patients were allowed to remain under blankets. Active warming was not allowed and patients had to lay on their back.

### Epidural anesthesia

Epidural anesthesia was performed according to a local protocol based on a loss of resistance method with saline 0.9%. Before insertion of the epidural catheter, all patients received intravenous access and routine hemodynamic monitoring. Patients were placed in a seated position while bending forward and rested on a pillow. After epidural catheter placement, patients were placed in supine position and a test dose with 2 ml lidocaine 1% was given. For the preoperative induction of the block, 8–10 ml of chirocaine 0.5% (AbbVie B.V. Hoofddorp, The Netherlands) was given as a bolus. During surgery, a continuous dose of chirocaine 0.5% was given through the epidural catheter. Supplemental analgesia and general anesthesia during surgery was standardized. To assess the effectiveness of the epidural block, the Numeric Rating Scale (NRS) for pain was measured in rest directly postoperative. Successful epidural anesthesia was defined as a NRS score ≤4. The use of postoperative opioids was measured as indicator of insufficient postoperative pain relief. The ear temperature was monitored during the preoperative and postoperative period (Medtronic Genius 2, USA).

### Thermal imaging

The changes in skin temperature were measured with an Ipad-add on (Apple Inc., Cupertino, USA) miniature infrared thermal imaging camera (FLIR ONE; FLIR systems, Inc., Wilsonville, USA). Non-contact skin temperature measurement is based on the emitted infrared radiation pattern of the skin or tissue, the radiation depends on the temperature of the object. The infrared light coming from the object is focused onto an infrared detector. For image processing, the detector sends information to sensor electronics to translate data into an image. The infrared image is transformed into a radiometric image by complex algorithms to be able to read temperature values from that image. The thermographic camera produces a temperature image with a resolution of 160 × 120 pixels. The camera was held approximately one meter away from the subject. All the values are color encoded. Temperatures are presented with a 1 °C interval. Thermograms were stored on the tablet for off-line analysis with Vernier thermal imaging software.

### Cold sensation test

The cold sensation test is part of standard practice and was performed directly after the thermographic measurement at t = 15. An ice cube was used to perform the cold sensation test. The response of the patient was classified as cold sensation or no cold sensation. A cold sensation test was scored positive when there was no cold perception in the blocked dermatomes, and negative when a perception of cold existed.

### Study design

**Fig. 1:**
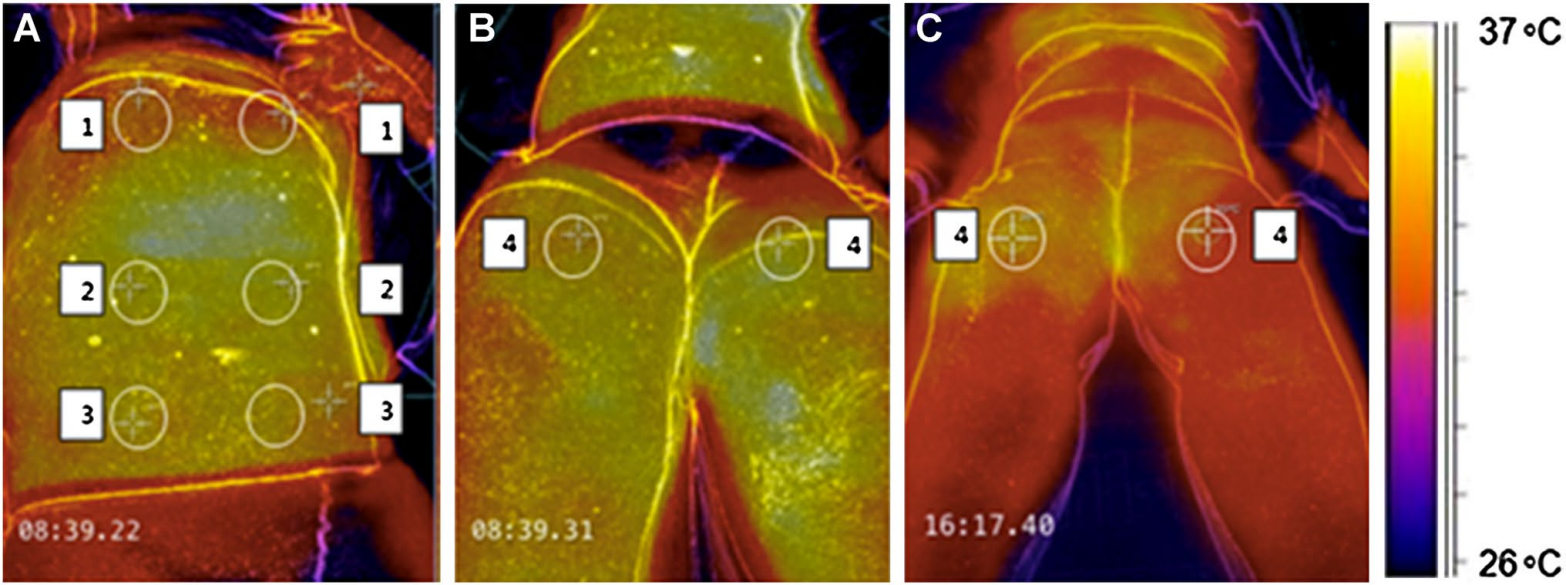
Examples of thermographic images of the anterior part of the abdomen and upper legs.* Panel A* and* B* show four different regions that were chosen to evaluate temperature differences.* Region 1*: hypochondriac region (*left* and *right*);* Region 2*: lumbar region (*left* and *right*);* Region 3*: iliac region (*left* and *right*);* Region 4*: the anterior thigh (*left* and *right*). * Panel C* shows a thermal picture taken 20 min after the epidural block. The right thigh is *brighter in color* than the left thigh, indicating that the right thigh (36 °C) has a higher temperature than the left thigh (35 °C)

One investigator performed thermographic imaging and the cold sensation test. In the first 15 min after insertion of the epidural catheter, five pictures of the skin of the blocked dermatomes were made with the thermographic camera. T = 0 has been taken before the local anesthetic was given. Patients were required to uncover the skin of the blocked dermatomes for 30 s before taking the picture to adjust to the surrounding temperature. Figure 1 shows typical examples of a thermal image. Panel A and B show four different regions to provide for uniform and quantitative temperature measurements. Region 1: hypochondriac region left and right. Region 2: lumbar region left and right. Region 3: iliac region left and right. Region 4: the anterior thigh left and right. Panel C shows a thermal picture after a local anesthetic has been given through the epidural catheter. The right thigh is brighter in color compared to the left tight, indicating that the right thigh has a higher temperature (36.0 vs. 35.0 °C).

Time zero (t = 0) was defined as the time before injection of the local anesthetic through the epidural catheter. Thermographic images were repeated at 5-min intervals (t = 0, 5, 10 and 15) until 15 min after administration of epidural anesthesia. To provide a quantitative measurement of the thermographic imaging and to assist in the interpretation of the color images, the thorax, abdomen and upper legs were divided in eight regions and the temperature of each region was measured (Fig. 1). Patient demographics, including age, gender, body mass index (BMI) and ASA classification were all recorded. All images where made at the same distance from the patient.

### Statistical analysis

Data were analyzed using Graphpad Prism 7 (Graphpad Software Inc., San Diego, California) and SPSS Statistics version 22.0 (IBM, New York, USA). Results were tested for normality using the Kolmogorov–Smirnov test. Data are presented as means and 95% confidence intervals (CI) if distributed normally. For patient characteristics a Mann–Whitney-U test was used. Comparisons of temperature changes from baseline were made by a Student’s t-test, corrected for multiple comparisons. Between group differences were calculated using a Chi square test. A P-value of <0.05 was considered significant.

To calculate the sensitivity and specificity of thermography for the detection of epidural success, a receiver operating characteristics curve (ROC) analysis was performed. To determine a cut-off point for calculating the sensitivity and specificity, multiple ROCs were performed where the temperature differences were dichotomized into less or more than 1, 2, 3 or 4° of difference in comparison to T = 0. The cut-off points were defined by the different areas under the curve. The temperature differences were compared with the NRS-score in order to calculate the specificity, sensitivity, positive predictive value and negative predictive value.

Sensitivity measures the proportion of positives that are correctly identified as such (e.g., the ratio of the number of patients who have a significant change in temperature of the skin/a positive cold sensation test over the total number of patients with a clinically functioning epidural block). Specificity measures the proportion of negatives that are correctly identified as such (e.g., the ratio of the number of patients without a significant temperature rise/negative cold sensation test over the total number of patients without a clinically functioning epidural block). Positive predictive value indicates the amount of patients with a positive test and who are correctly diagnosed. Negative predictive value indicates the amount of patients with a negative test and who are correctly diagnosed. To investigate for predictors that had an influence on the outcome of post-operative pain, a logistic and linear regression was conducted.

## Results

From the total study population of 64 patients, 3 patient were excluded due to no epidural anesthesia (n = 1) and procedural changes (n = 2). The study population consisted of 37 male and 24 female patients aging 67 ± 11 years. The majority of patients underwent a (hemi)colectomy (37.9%) or sigmoid/rectal resection (31.0%).

Fifty-five patients reported a NRS ≤4 (successful block; 90%) upon admission to the post-anesthesia care unit with a median NRS of 0 (0–1). There were no differences in demographic parameters between patients with a successful block and unsuccessful block (Table 1). Patients with a successful block received less opioid support during surgery than patients with an unsuccessful block (8.1 ± 5.6 vs. 13.3 ± 4.1 mg/kg/hr, respectively; P = 0.039). No differences were found between preoperative and postoperative temperature values measured in the ear (35.8 ± 1.6 vs. 35.9 ± 1.9 °C).

**Table 1:** Patient characteristics of the study group (n = 61)

	Overall (n = 61)	Successful (n = 55)	Unsuccessful (n = 6)
Age (years)	67 ± 11	65 ± 14	65 ± 16
Gender (M:F)	37:24	25:15	4:2
BMI (kg/m^2^)	25.6 ± 5.4	24.8 ± 3.1	25.2 ± 4.6
Median ASA	2 (2–2)	2 (2–2)	2 (2–2)

Data are represented as mean ± SD, frequency or median with interquartile range

*BMI* body mass index, *ASA* American Society of Anesthesiologists

**Fig. 2:**
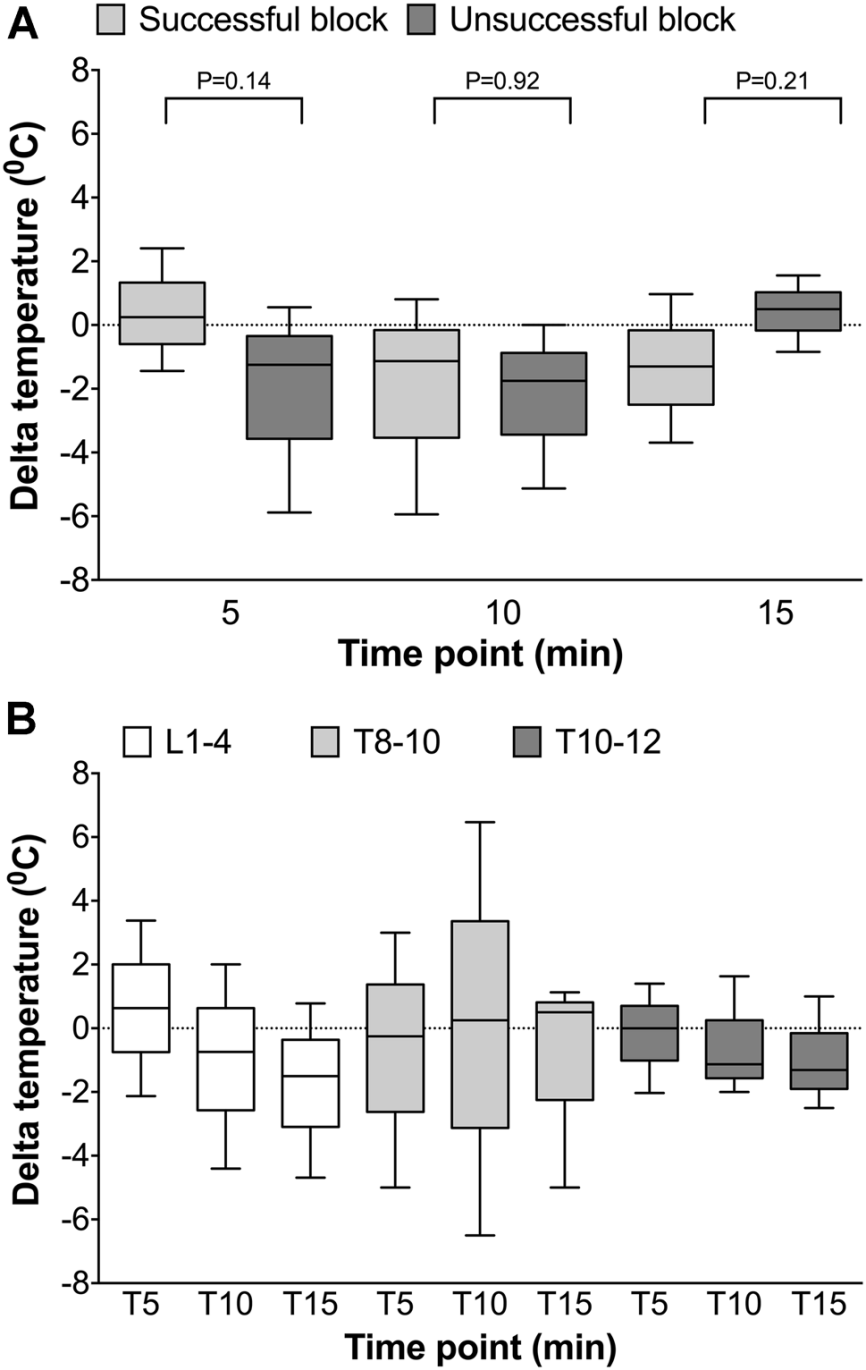
*Panel A* shows the temperature difference between baseline (T = 0) and T = 5, T = 10 and T = 15 for patients with a successful block (NRS <4; *light grey*) or unsuccessful block (NRS ≥4; *dark grey*).* Panel B* represents the temperature change over time for the epidural location lumbar 1–4, thoracic 8–10 and thoracic 10–12. *Boxes* represent medians with interquartile ranges. P-values are indicated in the figure

Figure 2a shows the temperature differences compared to baseline (T = 0) at 5, 10 and 15 min following epidural anesthesia. In patients with a successful block (NRS ≤4), a temperature drop was observed after 10 min following epidural anesthesia, and this drop remained until T = 15. In patients with an unsuccessful block, there was an initial drop in temperature that returned to 0 °C at 15 min following epidural anesthesia. There were no differences in the delta temperature between groups.

Figure 2b shows the change in temperature for an epidural location at L1–4 (white boxes), T8–10 (light grey boxes) and T10–12 (dark grey boxes). While the temperature decreased over time for L1–4 and T10–12, there was no clear direction in temperature changes for patients with an epidural at T8–10. The unsuccessful blocks were only present in patients with an epidural at L1–4 (4 patients) and T10–12 (2 patients).

To determine a cut-off point for the calculation of the sensitivity and specificity of thermography for the successfulness of epidural anesthesia, multiple ROC analyses were performed (Table 2). The highest AUC was retrieved at 15 min following epidural anesthesia, with a cut-off of 2 °C (AUC = 0.602). The AUC of the cold sensation test at 15 min following epidural anesthesia was 0.388.

**Table 2:** Area under the curves (AUCs) of the receiver operating characteristic (ROC)

	1 °C	2 °C	3 °C	4 °C
AUC 5 min after T = 0	0.569	0.555	0.518	0.494
AUC 10 min after T = 0	0.390	0.388	0.352	0.352
AUC 15 min after T = 0	0.569	0.602	0.553	0.528

Area under the curve (AUC) of the ROC analysis for temperature differences compared to baseline at 5, 10 and 15 min following epidural anesthesia. AUCs were used to determine the cut-off point for the sensitivity and specificity of thermography to determine the successfulness of epidural anesthesia

Table 3 shows the sensitivity, specificity and the positive and negative predictive value for the different measurement time points for thermography and the cold sensation test. Although the sensitivity of thermography was lower compared to the cold sensation test at 15 min following epidural anesthesia (0.54 vs. 0.97, respectively), the positive predictive value of both methods was comparable. The specificity of thermography at 15 min following epidural anesthesia was higher compared to the cold sensation test (0.67 vs. 0.25, respectively). However, the negative predictive value of thermography was low (0.17).

**Table 3:** Predictive value of thermography and the cold sensation test for the successfulness of the epidural block

	Sensitivity		Positive predictive value	
	Thermography	CST	Thermography	CST
5 min after T = 0	0.61		0.89	
10 min after T = 0	0.61		0.83	
15 min after T = 0	0.54	0.97	0.92	0.93

	Specificity		Negative predictive value	
	Thermography	CST	Thermography	CST
5 min after T = 0	0.50		0.15	
10 min after T = 0	0.17		0.06	
15 min after T = 0	0.67	0.25	0.17	0.50

Sensitivity, specificity, positive predictive value and negative predictive value for thermography and the cold sensation test at 5, 10 and 15 min following epidural anesthesia. Time difference is expressed in minutes

*CST* cold sensation test

## Discussion

The present study investigated whether thermography can be used as alternative method for assessing the efficacy of epidural anesthesia when compared to the cold sensation test. We found that thermographic measurements are an objective method to assess the effectiveness of an epidural block. However, in contrast to previous reports focusing on regional anesthesia techniques, epidural anesthesia resulted in a temperature drop rather than a temperature increase of the skin [16, 17]. We found that the positive predictive value of thermography is equal to the positive predictive value of the cold sensation test. However, despite a higher specificity when compared to the cold sensation test, the negative predictive value of thermography was very low. This implies that thermography is reliable when epidural anesthesia is successful, but unreliable with respect to the diagnosis of unsuccessful epidural anesthesia. In these cases, a combination of thermography and the cold sensation test could be considered as valuable alternative.

Our thermography data show a consistent decrease in skin temperature in case of successful epidural anesthesia. These observations are however in contrast to previous studies in patients undergoing a local blockade. In the study of Galvin and colleagues, an axillary block for hand surgery induced a temperature increase of the forearm skin of 5 °C in case of a successful block. This temperature increase was almost ignorable in patients with an unsuccessful block [16]. Klaessens et al. showed that thermography revealed differences in skin temperature differences that corresponding with the areas of the neuraxial block [17]. In a study focusing on the efficacy of thermography to assess the successfulness of an infraclavicular brachial plexus block, a temperature increase of 3 °C was observed in the forearm [18].

In 1994, Cheema and colleagues described a case report in a pediatric patient where the effect of epidural anesthesia at T7/T8 was assessed by thermography [19]. In agreement with our findings, they also showed a slight decrease in skin temperature. In a second study in six adult patients the same authors demonstrated that preblock skin temperature decreased particularly in the cervical dermatomes, the thoracic dermatomes T4–T8 and T10–T12 and the lumbar dermatomes L1–L3 [20]. In agreement with the observations of Cheema, we also observed a temperature decrease in the dermatomes corresponding with L1–L4 and T10–12, while the changes in temperature corresponding with a blockade at T8–10 were highly variable. Similar findings were presented by Freise and colleagues, showing that the decrease in skin temperature assessed by thermography was particularly observed in T10–12 [21].

Epidural anesthesia reduces central autonomic thermoregulatory control, leading to periprocedural hyperthermia [22, 23]. The decrease in temperature during epidural anesthesia is mainly attributed to a core-to-peripheral redistribution of body heat [24, 25]. Additionally, epidural anesthesia increases subcutaneous tissue oxygenation due to vasodilation [26]. Both physiological mechanisms will contribute to the decrease in skin temperature as observed in our study.

Liquid substances such as gel or disinfection spray on the skin surface an influence infrared imaging results. Bernard et al. state that the surface temperature needs to be corrected when solutions like disinfection or gel are being put on the skin. In the case of this study the disinfection was administered on the back, while the thermographic pictures were measured on the clean skin of the abdominal area. Disinfection was only administered in the area of interest after the last picture was made, the temperature thus needed no correction. Moreover Bernard et al. also found that clean skin has a minimum reflection of ambient temperature due to its high emissivity [27].

We performed a ROC analysis to calculate a positive and negative predictive value for thermography as assessment for the successfulness of epidural anesthesia. The comparative method was the cold sensation test, which is subjective, time consuming, depending on patient cooperation and may be associated with false positive and false negative results [6, 7]. With respect to the detection of patients with a successful block, thermography was equivalent to the cold sensation test. However, the cold sensation test was superior in the detection of patients with suboptimal epidural anesthesia. While the latter category is crucial in the strategy to reduce postoperative pain, we here conclude that thermography cannot replace the cold sensation test for the assessment of the successfulness of epidural anesthesia. Our findings are in contrast to the findings of Minville et al. showing that the infrared thermometer is a reliable and early indicator to detect a successful infraclavicular branchial plexus block [18]. However, the rise in skin temperature that is associated with a local block and the absence of a ROC analysis in this publication limit a comparison with our findings [18]. Our analyses show the highest positive predictive value at 15 min following induction of epidural anesthesia. At this time point, patients with a successful block showed a persistent decrease in skin temperature, while the skin temperature in patients with an unsuccessful block returned to baseline values. This is in accordance with the known onset time of levobupivacaine, which is approximately 15 min [19].

The definition of a successful block was influenced by the administration of opioids during the procedure. Patients with an unsuccessful block received more opioids per hour than patients with sufficient epidural anesthesia. Other parameters, including age or the duration of surgery showed no relationship with postoperative pain.

There were limitations in this study. The thermographic camera is easily usable and could be attached to your smartphone or tablet, but the application used needs improvement for use in daily clinical practice. Temperatures were measured only in whole degrees instead of one-tenth of a degree. This low resolution reduces the ability to distinguish a change in temperature. Moreover, general anesthetics, such as propofol and sufentanil, also have an effect on dilatation of the skin vasculature, and hence influence the skin temperature. As a consequence, thermographic measurements were stopped upon induction of general anesthesia.

Core temperature was measured in the ear with infrared thermometry. However, accuracy of infrared ear thermometry is limited.

Another limitation of this study is the time chosen for the skin to adjust to the surrounding temperature prior to examination. This period of 30 s might be too short for the skin to adjust completely to its surrounding temperature. Lastly, we only performed the cold sensation test at 15 min after administration of the epidural anesthetics, so there is only one time point where the cold sensation test could be compared with the thermography.

We conclude that thermographic temperature measurements are instantly available, easy to perform, objective method that will provide additional information to the cold sensation test in the assessment of the effectiveness of an epidural block. Further studies are required to assess the use of thermography to evaluate the effectiveness of epidural anesthesia, with emphasis on the differences between skin temperatures among distinct dermatomes.
